# The Consequences of Performance Measurement Systems in Multilevel Governance Health Systems: The Case of the Italian National Healthcare Service

**DOI:** 10.34172/ijhpm.9384

**Published:** 2026-06-13

**Authors:** Federico Rotondo, Lucia Giovanelli, Nicoletta Fadda, Russell Mannion

**Affiliations:** ^1^Department of Humanities and Social Sciences, University of Sassari, Sassari, Italy.; ^2^Department of Economics and Business, University of Sassari, Sassari, Italy.; ^3^Health Services Management Centre, School of Social Policy, University of Birmingham, Birmingham, UK.

**Keywords:** Performance Measurement System, Multilevel Governance, Functional Consequences, Dysfunctional Consequences, Effectiveness Factors, Italy

## Abstract

**Background:**

Performance measurement systems (PMSs) have become an essential component of health system reforms globally and are increasingly used to assess, reward and improve provider performance. While PMSs can be valuable tools for achieving desired health outcomes, their effectiveness depends on how they are designed, implemented, and used within complex multilevel governance structures. There is evidence that PMSs may also have unintended negative consequences. Drawing on new empirical evidence, the present study examines the consequences of PMSs implemented in multi-level governance systems through the analysis of the Italian National Healthcare Service (INHS), which exemplifies a decentralised health system with multiple levels of governance.

**Methods:**

The study employed a mixed methods approach combining quantitative and qualitative methods to examine the functional and dysfunctional consequences of PMSs in the INHS across three governance levels: (*i*) State-Regions, (*ii*) Regions-healthcare organisations, and (*iii*) Healthcare organisations-healthcare professionals.

**Results:**

We identified three key functional consequences. First, PMSs drive improvement by facilitating benchmarking, advocacy, and collaboration. Second, quantifying activities and services improves objectivity and transparency. Third, national PMSs provide a comprehensive view of performance across multiple dimensions and provide a more holistic understanding of how different aspects of the system interact. We confirmed previously reported dysfunctional consequences of PMSs found in other health systems and identified three new dysfunctional consequences in the INHS: Measurement Overload, Misconsideration, and Exploitation.

**Conclusion:**

Based on our analysis and existing literature, we propose ten key factors for strengthening performance measurement in the INHS. While this study offers novel evidence on the functional and dysfunctional consequences of PMSs in the Italian system, our research is context-specific, and the applicability of these factors to other multi-level health systems remains an area for future empirical testing.

## Introduction

**Key Messages**

**Implications for policy makers**

- At the national level, policy-makers should create better-tailored incentives to enhance motivation at all levels of the health system.
- At the regional level, policy-makers should integrate national indicators into the evaluations of healthcare organisations to enhance alignment and ensure system wide consistency.
- At the organisational level, policy-makers should go solely beyond financial incentives and use different evaluation methods to motivate professionals.
- Prioritise the integration and interoperability between all levels to facilitate the timely and seamless flow of performance information and data exchange.
- Deploy tailored support and remediation measures for underperforming Regions and providers should be implemented to achieve sustainable improvement.

**Implications for the public**

This research investigates how performance measurement systems (PMSs) impact healthcare systems with multiple levels of governance, focusing on functional and dysfunctional consequences. The study aims to improve relationships between different government levels and strengthen performance management in healthcare. Ultimately it aims to benefit the public by fostering a stronger performance culture in the Italian National Healthcare Service (INHS) and increased engagement with stakeholders. A key element for comprehensive performance assessment is the incorporation of citizen and patient perspectives. To prevent trust erosion caused by poor local performance or misinformation, we suggest proactive communication strategies and training for both the public and healthcare professionals. This would mean sharing performance information in plain, jargon-free language and taking steps to improve health literacy among the general public.

Performance measurement systems (PMSs) have become a cornerstone of health system reforms globally. These systems, initially implemented under New Public Management principles, aim to drive improvements in healthcare delivery and patient outcomes by enhancing efficiency, effectiveness and quality while promoting accountability and transparency.^1,2^ Healthcare systems are often managed at multiple levels (eg, national, regional, and local), which creates a hierarchical governance structure with complex networks of agency relationships with each level of the hierarchy and stakeholder group (eg, policy-makers, regulators, commissioners, and healthcare providers) often having potentially divergent motivations and information needs about healthcare performance.^3^ This multi-level structure and diverse stakeholder landscape can create inherent challenges for effective performance measurement and improvement. Although the benefits and the unintended and dysfunctional consequences of PMS in healthcare are well documented,^4-6^ empirical research inmultiple-level governance health systems is scarce. Against this background, the present study focuses on the Italian National Healthcare Service (INHS), which represents a prime example of a highly decentralised system with multiple levels of governance, where roles and responsibilities are distributed across national, regional, and local authorities, providing an ideal setting to explore the consequences of PMSs implemented in multi-level governance systems. To the best of our knowledge, this study provides the first comprehensive analysis to explore the consequences, in terms of challenges and opportunities, arising from PMSs within a multi-level governance healthcare system, using the INHS as a specific case study.

###  Multilevel Governance and Performance Measurement Systems in Healthcare 

 Multilevel governance involves the strategic sharing and coordination of policy, financial resources and decision-making power across different levels of government and multiple stakeholders.^7,8^ Due to its inherent complexity, healthcare governance is often distributed across multiple levels.^9^ From global multilateral organisations, such as the World Health Organization (WHO), to individual healthcare providers. Supranational global organisations such as the WHO play a crucial role in setting global health standards, coordinating cross-border efforts, and responding to global health emergencies. National governments establish policies, funding, and regulations that define the healthcare system within their borders. Regional and local governments often organise service delivery, allocate resources, and coordinate local authorities and provider organisations. Finally, individual healthcare professionals, including nurses and doctors and other specialists, are the primary actors ultimately responsible for delivering care to patients.^10^

 Agency theory, which analyses the relationship between a principal and an agent,^11^ offers a useful framework for understanding how power and responsibility are distributed across different levels of government. It examines how central authorities (principals) delegate tasks and decisions to local actors (agents). In health systems, the principal could be a central authority such as a government ministry or a regional health authority, while the agent could be a local healthcare provider such as a hospital.^12^ This relationship often faces the principal–agent problem, which arises when the agent and the principal have divergent motivations and information asymmetries allow agents to prioritise their own interests over the principal’s goals. Such misalignments can lead to persistent conflicts and operational inefficiencies as authority flows through multiple bureaucratic tiers. The agent may prioritise their interests (for example, maximising their income) over the principal’s goals (for example, providing high-quality care). Increasing layers of delegation can intensify the principal-agent problem.^13^ As the number of delegated decision-making layers increases, information asymmetry tends to increase, making it harder for the principal to monitor the agent’s actions and ensure alignment with their interests. This can also lead to increased transaction costs as the principal is forced to invest in complex monitoring mechanisms to audit the agents’ actions.^14^ PMS can play a crucial role in mitigating the principal-agent problem by monitoring an agent’s actions and ensuring accountability for outcomes.^15^ A variety of incentives (rewards and sanctions) have been attached to the use of performance measures in healthcare.^16^ These can range from financial (eg, payment for performance schemes) to non-financial, such as increased provider autonomy or enhanced organisational reputation.^9,17^ By linking rewards or sanctions to specific performance metrics, principals can encourage agents to act in ways that align with the principal’s interests. While incentives can be effective, they can also lead to unintended and dysfunctional consequences.

 PMSs are managed feedback processes comprising three sequential stages: (*i*) Measurement: the systematic collection of performance using various instruments and tools; (*ii*) Analysis: the interpretation of data to identify trends, patterns and performance insights; and (*iii*) Action: the implementation of appropriate actions taken to drive improvement, involving actions to address underperformance and reinforce areas of success.^12^ When properly designed and implemented, PMSs can align the incentives and motivations of principals and agents and consequently improve performance.^5^ Motivations for using PMSs in healthcare may differ for governance levels. These range from increasing accountability and fostering market mechanisms to improving service quality and generating public health data. The specific goals typically align with the hierarchical level within the healthcare system.^1^

 To gain a comprehensive understanding of healthcare performance, a multidimensional approach is essential.^18-20^ While PMSs in healthcare must be comprehensive to capture all relevant aspects of performance, they must also be flexible enough to address the unique characteristics of different healthcare systems and providers.^21-23^ Empirical evidence suggests that any approach to healthcare performance measurement may work for some health services, some of the time, but no single approach is likely to be a panacea.^24^ For example, what works for primary care may not work for surgical specialities, and PMSs may be more suitable for periods of stability compared to times of rapid structural change. By unravelling the specific contexts where various approaches succeed or fail, designers can create more sophisticated PMS design that maximises positive outcomes while minimising dysfunctional consequences.^25^ A number of empirical studies have examined the factors associated with effective PMSs. Smith identifies four key success factors^4^: the ability of measures to reflect the system’s objectives, data quality, the incentives for the subject to be evaluated, and the organisation’s culture. Data quality, specifically its availability, completeness and reliability, presents significant challenges for PMSs.^26^ Ensuring these aspects of data appear to be crucial for informed decision-making, accurate analysis, and effective strategies.^27-30^ Efforts to enhance data quality for PMS include the development of better integrated health information systems and new technological infrastructure,^31^ the incorporation of qualitative data,^32^ and the provision of organisational support.^33^ Organisational culture can also increase or hinder the acceptance and use of performance information,^34^ and there is a key role for senior leaders and managers in fostering a culture that values and effectively uses performance data. Recent studies have emphasised the critical role of stakeholder engagement in PMSs, throughout the design, implementation and evaluation of PMSs in healthcare,^35,36^ Actively involving healthcare professionals and administrative staff enhances transparency and accountability,^37,38^ while engaging general practitioners and citizens,^39^ helps guide choices and develop people centred approaches.^21,40,41^ Stakeholder involvement has also been viewed as an effective way of tackling poor data quality.^42^

 A range of dysfunctional consequences has emerged following the introduction of PMSs in the public sector.^43^ This has been referred to as a performance paradox – a counterintuitive phenomenon where an increased focus on performance metrics can lead to unintended negative consequences or a decline in actual performance.^44^

 While quality measurement has great potential to enhance the quality of care, Casalino suggests it may undermine physician professionalism by increasing the burden of adhering to core principles.^45^ This tension is further explored by Kerpershoek et al,^46^ who found that Dutch medical professionals’ responses to PMSs were often motivated by a desire to protect their professional ethos. Freeman conducted a systematic review of the use of performance indicators to improve healthcare quality in the UK, reporting major problems, including their potential to undermine the conditions required for quality improvement and to create perverse incentives, as well as technical issues such as data availability and reliability.^26^ Conrad and Guven Uslu employed a case study approach to investigate the impact of a new PMS imposed by regulatory bodies on English hospital Trusts, highlighting problems arising from the tool’s instrumental design and use.^24^

 Several useful typologies and frameworks have been developed to classify the unintended and dysfunctional consequences of PMS in healthcare.^6,26^ Powell et al identified three key negative outcomes in primary care^47^: inappropriate clinical care, diminished attention to patient concerns and weakened patient education and autonomy. Notably, these issues often arose from local implementation strategies designed to meet national mandates. Similarly, Aryankhesal et al identified seven dysfunctional consequences of Iran’s hospital grading system.^48^ Li and Evans conducted a rapid review, followed by semi-structured interviews with administrators and clinical leads involved in cancer and renal care in Ontario, to develop a comprehensive typology of the unintended consequences of PMS.^6^ They identified two novel unintended consequences for organisations and providers: increased perceived injustice due to social comparisons and toxic ambition. Both studies drew on Mannion and Braithwaite, who identified 20 different dysfunctional consequences of the UK’s national PMS.^2^ Their typology classifies dysfunctional consequences into four broad categories: poor measurement (Measurement Fixation, Tunnel Vision, Myopia, Ossification, Anachronism, and Quantification Privileging), misplaced incentives and sanctions (Complacency, Silo-creation, Overcompensation, Undercompensation, Insensitivity, and Increased Inequality), breach of trust (Misrepresentation, Gaming, Misinterpretation, Bullying, Erosion of trust, and Reduced staff morale), and politicisation (Political Grandstanding and Creating a Diversion).

###  The Italian National Healthcare Service 

 The INHS, established in 1978, is a tax-funded universal system which guarantees the provision of comprehensive healthcare services to the entire population. The art. 117 of the Constitution assigns health protection to the concurrent competence of the State and the Regions, creating a three-tiered structure: the upper national level, managed by the central government through the Ministry of Health, the middle regional level, managed by the 19 Regions and the two Autonomous Provinces of Trento and Bolzano, and the lower operational level comprising healthcare organisations, ie, local healthcare authorities (LHAs), hospitals and Scientific Institute for Research, Hospitalisation and Healthcare (IRCCS).

 Since 2000, the Conference of State-Regions has become the main body for negotiating health policy, with the central government providing the legislative framework for healthcare and setting the essential healthcare levels (Livelli Essenziali di Assistenza or LEA) that the Regions must deliver, also allocating a dedicated budget for the purpose. This funding allocation, however, applies only to 16 of Italy’s 21 Regions, specifically excluding three special-statute Regions (Friuli-Venezia Giulia, Sardinia, and Aosta Valley) as well as the two autonomous provinces. In 2000, a mechanism to control the effectiveness and efficiency of LEA provision by the Regions was introduced (Sistema di Garanzia). From 2008 to 2019, the Permanent Committee for the Assessment of LEA provision used a standardised set of quantitative performance indicators (the so-called Griglia LEA) for its assessment. In 2020, the Griglia LEA was superseded by the Nuovo Sistema di Garanzia (NSG), which introduced 88 indicators, grouped into prevention, district care, hospital care, four context indicators, one indicator of social equity, and 10 indicators for monitoring six diagnostic-therapeutic care pathways (Percorsi Diagnostici Terapeutici Assistenziali or PDTA). The NSG, which provides a financial incentive through its CORE subset of 22 indicators, is used to evaluate the provision of LEAs and permit Regions to have access to a financial reward (0.50% of the unique fund in 2023).

 The national government also monitors the financial sustainability of the regional systems prescribing “recovery plans” (RPs) (Law no. 311/2004) (mandatory three-year agreements between the State and the Regions) in the event of a structural deficit exceeding 7% of the regional health fund. RPs require Regions to commit to assuring the LEAs, as well as restoring a financial balance. Furthermore, excessive structural deficits, failure to implement RPs, disrespect of LEAs or extraordinary circumstances (eg, criminality) can lead the State to intervene and place Regions under the administration of an external commissioner (receivership). Thus, this governance model incentivises regional autonomy by linking performance directly to the retention of administrative control.

 In 2010, the National Agency for Regional Health Services (Agenas) introduced the “Programma Nazionale di Valutazione degli Esiti” (PNE), as a PMS to monitor healthcare outcomes across Italian hospitals (1363 in 2024). The PNE benchmarks all Italian public and private hospitals against about 200 outcome or process indicators and functions mainly through reputation incentives, as it is not linked to any financial reward but still has a significant impact on organisational and professional standing. The primary goal of PNE is not merely to rank hospitals, but to incentivise quality audits and the implementation of targeted improvement strategies.^49^ The Regions maintain considerable autonomy over the organisation and delivery of healthcare services. However, they must operate within strategic guidelines established at the national level. Therefore, each Region develops its own unique PMS to plan and monitor healthcare services provided by LHAs and hospitals within its jurisdiction. On an annual basis, Regions then assign specific performance objectives to the General Directors of these healthcare organisations to ensure alignment with broader strategic goals.

 At the operational level, healthcare organisation translates national and regional objectives into actionable local goals for individual departments, units and teams to ensure service quality, care appropriateness, and cost containment. Within a multilevel system like the INHS, PMSs effectively mitigate principal-agent problems when two conditions are met, primarily in the State-Region relationship. The first condition is that the objectives underlying the respective PMSs are aligned; the second is that robust incentives are in place to steer behaviours toward system-level priorities.

Figure 1 schematically depicts the PMSs and multi-level governance in the INHS.

**Figure 1:**
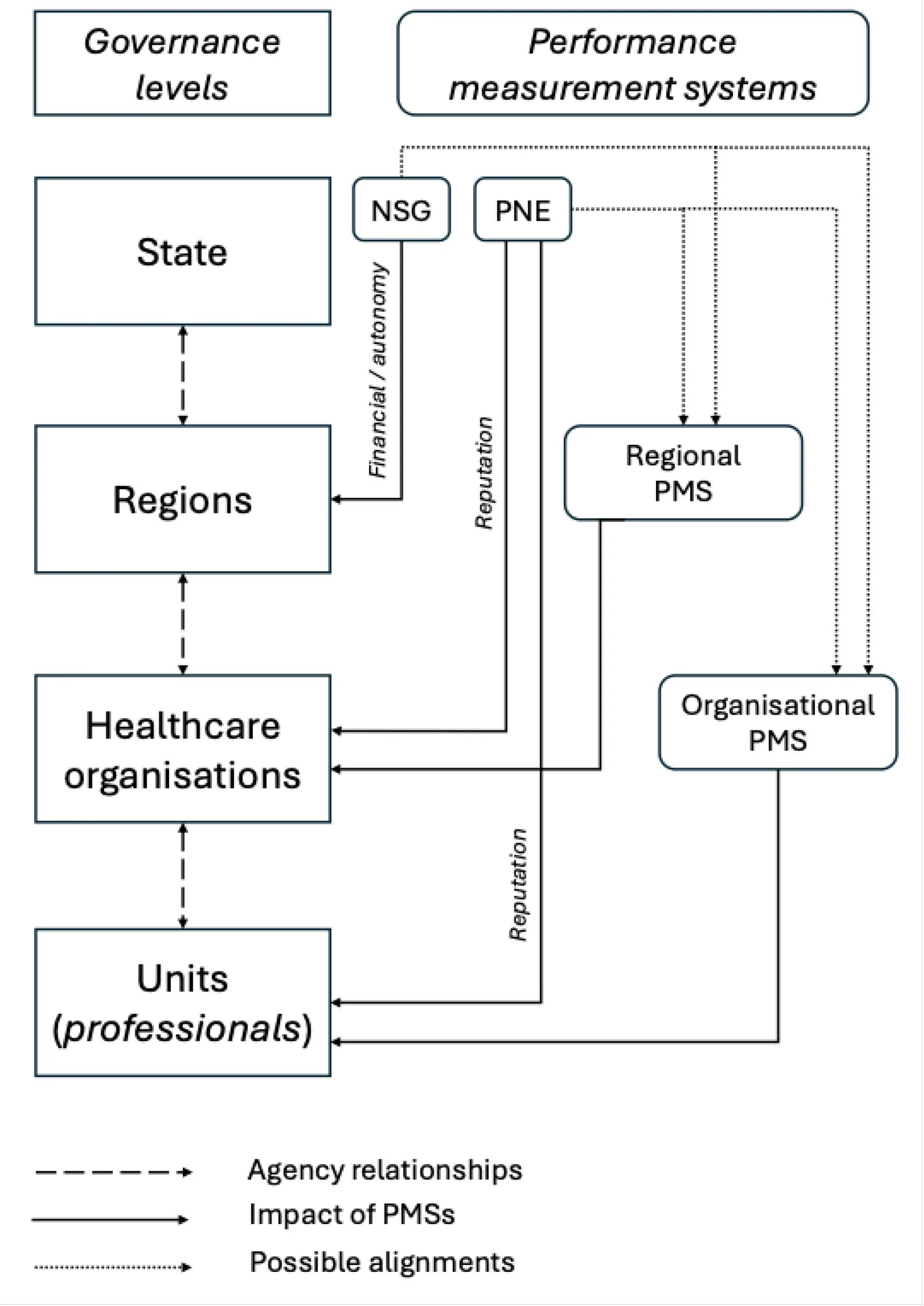
Performance Measurement Systems and Multi-level Governance in the Italian National Healthcare Service. Abbreviations: PMS, Performance measurement system; NSG, Nuovo Sistema di Garanzia; PNE, Programma Nazionale di Valutazione degli Esiti.

## Methods

 The study employed a mixed methods approach combining quantitative and qualitative methods across two sequential phases. The first phase, called the “quantitative approach,” represents a preliminary yet essential phase for a thorough analysis of the consequences of PMSs in multilevel governance health systems. It was, in fact, intended to verify the degree of alignment between national and regional PMSs’ goals and assess the effectiveness of national incentives in reducing agency problems as outlined earlier in this article. This initial analysis served to identify the Regions and health organisations for subsequent qualitative exploration, and ensured the accurate interpretation of emerging data.

 The first phase, from January to July 2024, consisted of two stages:

- Stage 1.1: A review of national and regional legislation and official documents to assess alignment between regional and national PMSs.
- Stage 1.2: Involved a desk analysis of existing data and documents to assess regional performance in the national PMSs and to understand how different incentives may have influenced outcomes. To compare regional performance across the two national PMSs (NSG and PNE), two composite indicators were developed using a benchmarking approach. This information enabled mapping regional performance alongside the two national PMSs and informed the selection of Regions for further in-depth qualitative investigation (Stage 2.2).

The second phase, called the “qualitative approach,” was conducted from July 2024 to May 2025 and explored the motivations, experiences, and perspectives on the positive and negative impacts of PMSs at multiple levels within the INHS. Participant insights and key informant testimonies were interpreted alongside empirical evidence from the first research phase. This phase employed various qualitative methods across three stages, focusing on national, regional and organisational levels. See Table 1 for a detailed summary.

**Table 1:** Methodological Details by Phase and Stage

**Quantitative Approach**				
**Stage – Period**	**Aim**	**Source**	**Details**	
1.1. January to May 2024	Assessing the alignment of aims between the regional PMSs and the national PMSs	Current legislation and official documents of the national and regional PMSs over the period 2019-2023	The analysis covered all Italian Regions and Autonomous Provinces. The resolutions of the regional governments, strategic plans and guidelines (when existing), and RPs were downloaded from the official websites (a total of 182 documents). The document analysis of regional PMSs focused on the following aspects: • Explicit reference to national PMSs; • Weights of health service indicators and administrative/financial indicators; • Weights of NSG indicators and NSG CORE indicators; • Weight of PNE indicators.	
1.2. June to July 2024	Understanding how different national incentives influence regional performance	National PMSs: NSG and PNE reports over the period 2016-2023	NSG – *Incentives: financial and autonomy* A composite indicator was developed using a benchmark approach. First, we built an average overall performance indicator, obtained by adding up performances achieved in hospital, prevention and district care, over the period investigated, for each Region. Second, we calculated a benchmark score with the following formula: NSG benchmark score = Region’s performance/Best in class performance	PNE – *Incentive: reputation* First, since it assesses outcomes of hospitals, it was decided to build a single score at a regional level. Second, it was decided to focus on the 20 included in the Treemap, which is the synthetic evaluation tool covering all clinical areas used by Agenas. Five district indicators were then added, for a total of 25. Then, for each indicator, the average median over the period investigated was used. The following formulas were applied to calculate benchmark scores. For 21 indicators for which the lowest score was considered “best in class”: Benchmark score = (Worst in class - Region’s performance) / (Worst in class – Best in class performance) For four indicators for which the highest score was considered “best in class”: Benchmark score = Region’s performance/Best in class performance Finally, a composite indicator (PNE) was calculated by adding up the 25 benchmark scores, all of which are given the same weight, in the following way: $PNE_{j}=\frac{1}{25}+\sum_{i=1}^{m}d_{i}$ where PNE is the composite indicator for Region *j*, *d*_i_ is 1 if the Region is the “best in class” for indicator i (otherwise, it is equal to the benchmark score), and *m* is the maximum number of benchmark scores, which amounts to 25. The composite indicator was then transformed using the same “best in class” approach.
**Qualitative Approach**				
**Stage - Period**	**Focus**	**Source**	**Details**	
2.1. July to November 2024	National level	(a) Annual public presentations of official reports of NSG and PNE. (b) Semi-structured interview	(a) The two launch events were recorded and transcribed before analysis; (b) The Technical-Scientific Director of the PNE was interviewed.	
2.2. December 2024 to February 2025	Regional level	Semi-structured interviews with four senior executives from different Regions	The three criteria used for selecting the four Regions were: a) Performance achieved in the two National PMSs (NSG and PNE): one Region with high performance and one Region with low performance in both PMSs, one Region with a high performance in PNE and low in NSG, and one Region with a low performance in PNE and a high performance in NSG; b) A range of geographical locations across Italy; c) Regions with varying degrees of financial and decision-making autonomy.	
2.3. March to May 2025	Organisational level	(a) Semi-structured interviews to eight healthcare organisations from the four selected Regions, equally divided into LHAs and hospitals. (b) Four focus groups involving professionals	(a) General Directors were interviewed. (b) The focus groups were held at the end of April 2025 and were facilitated by two of the authors as part of mandatory management training programmes for healthcare professionals. Each group comprised 30 participants (120 in total), with 60% being physicians and 40% in management positions. The focus groups lasted on average 90 minutes and focused on the functional and dysfunctional consequences of PMSs across all levels on the daily work of professionals.	

Abbreviations: PMS, Performance measurement system; RPs, recovery plans; NSG, Nuovo Sistema di Garanzia; PNE, Programma Nazionale di Valutazione degli Esiti; LHAs, local healthcare authorities.

 Interviews followed a semi-structured format guided by a literature derived protocol consisting of four thematic sections: objectives, design stage, characteristics, and process. While applied across all governance levels, the guide was iteratively adapted with level-specific probes, resulting in 18 questions at the national level, 24 for the regional level, and 30 for the organisational level.

 To ensure validity and relevance, a pilot study was conducted in October 2024 with three practitioners who held key roles at each governance tier, and their feedback directly informed the final refinement of the questions. The interviews were conducted by three of the authors, either in person, online or via telephone. All participants provided informed consent for participation and consent in full compliance with General Data Protection Regulation (GDPR) 2016/679 EU and Italian Legislative Decree no. 196/2003. The interviews were transcribed verbatim, with an average duration of 60 minutes. Data were processed interactively between February and May 2025, using a systematic three-stage coding process: open coding to identify initial concepts, axial coding to establish relationships between categories, and a cross-case search to synthesise broader thematic patterns.

 The results are divided into two main sections corresponding to the research phases: quantitative and qualitative evidence. The former sheds light on the degree of alignment between national and regional PMSs and the effectiveness of national PMS incentives. The latter interprets these findings through interviewee perceptions to identify the positive and dysfunctional consequences of PMSs within the INHS multi-level governance system.

## Results

###  Quantitative Evidence

####  Alignment Between Regional and National Performance Measurement Systems

 These findings provide an overall view of the degree of alignment between the underlying aims of the national and regional PMSs. This alignment is a necessary condition, as it strongly influences the capacity to steer the activities of regional health systems, which are responsible for organising services and their outcomes, towards system-wide goals. Table 2 details, on a regional basis, the extent to which the regional PMS objectives explicitly reference national objectives, the weight of health-care indicators within the total, and the weight within the total of the indicators directly linked to the two main national PMSs, the NSG and the PNE.

**Table 2:** Alignment Between National and Regional Performance Measurement System

**No.**	**Regions/Provinces**	**Explicit Connection**	**Health Area Weight (%)**	**Administrative/Financial Area Weight (%)**	**Connection to NSG (Weight)**	**Connection to NSG CORE (Weight)**	**Connection to PNE (Weight)**
1	Abruzzo	Yes	26.50	73.50	17.83	16.58	3.08
2	Aosta Valley	Yes	15.00	85.00	15.00	15.00	15.00
3	A.P. of Bolzano	Poor	5.00	95.00	10.00	5.00	0
4	A.P. of Trento	Yes	23.00	77.00	3.50	1.50	1.50
5	Basilicata	Yes	63 (LHA), 67 (others)	37 (LHA), 33 (others)	51 (LHA), 19 (others)	27 (LHA), 6 (others)	11 (LHA), 21 (HO)
6	Calabria	Yes	56.00-57.00	44.00-43.00	59.00-56.00	49.00-52.00	12.00
7	Campania	Yes	12.50	87.50	12.50	12.50	3.41
8	Emilia-Romagna	Yes	75.83	24.17	23.33	14.17	19.16
9	Friuli-Venezia Giulia	Yes	77.02	22.98	30.91	26.36	8.18
10	Lazio	No	39.00 (LHA), 37.00 (HO), 30.00 (IRCCS)	61.00 (LHA), 63.00 (HO), 70.00 (IRCCS)	4.00 to 12.00	2.00 to 6.00	0
11	Liguria	Yes	49.00 (LHA) 35.00 (IRCCS)	51.00 (LHA), 65.00 (IRCCS)	18.37 (LHA), 8.75 (IRCCS)	11.37 (LHA), 8.75 (IRCCS)	0
12	Lombardy	Yes	38.50	61.50	7.00	7.00	0
13	Marche	Yes	48.39	51.61	19.58	15.89	4.32
14	Molise	Yes	65.00	35.00	35.00	31.00	12.00
15	Piedmont	Poor	24.50 (LHA) 32.20 (HO)	75.50 (LHA), 67.80 (HO)	11.50 (LHA), 11.20, 14.20 (HO)	11.50 (LHA), 11.20, 14.20 (HO)	13.50 (LHA), 18.20, 24.20 (HO)
16	Puglia	Yes	29.83	70.17	22.58 (LHA), 19.72 (HO)	19.58 (LHA), 16.01 (HO)	7.44 (LHA), 13.00 (HO)
17	Sardinia	No	9.50 (LHA)	90.50 (ASL), 100.00 (others)	7.00	4.50	0
18	Siciliy	Yes	52.00 (LHA), 43.33 (HO)	48.00 (LHA), 56.67 (HO)	27.00 (LHA), 21.00 (HO)	26.00 (LHA), 20.00 (HO)	20.00
19	Tuscany	Yes	40.10 (LHA), 36.76 (HO), 31.76 (IRCCS)	59.90 (LHA), 63.24 (HO), 68.24 (IRCCS)	18.60	14.40	19.17
20	Umbria	Yes	25.00	75.00	2.00	2.00	2.00
21	Veneto	Yes	38.16 (LHA), 35.60 (HO)	61.84 (LHA), 64.40 (HO)	10.33	8.88	5.29

Abbreviations: NSG, Nuovo Sistema di Garanzia; PNE, Programma Nazionale di Valutazione degli Esiti; LHA, local healthcare authority; HO, hospital; IRCCS, Scientific Institute for Research, Hospitalisation and Healthcare.

 In 17 out of 21 cases (81%), regional PMSs are explicitly linked to national PMSs. While 16 out of 21 Regions (76%) prioritise administrative/financial metrics over health, weights vary from 77% health focus in the Friuli-Venezia Giulia Region to only 5% in the Autonomous Province of Bolzano. Overall, considering the varying weights of indicators used to evaluate different types of healthcare organisations (eg, LHAs and hospitals), the average weights for health and administrative/financial indicators are approximately 38% and 62%, respectively. Analysis of regional PMSs reveals a significant focus on national indicators, with NSG indicators receiving the highest average weight (19.86%). Calabria exhibits the highest percentage (59%), along with Basilicata (LHAs only), the only regions exceeding 50%, while Umbria places only 2% weight on NSG indicators. On average, NSG CORE indicators account for 15.76%. Calabria again ranks highest for NSG CORE indicators (52%), and Umbria the lowest (2%). Overall, Regions under LEA assessment assign greater weight to NSG indicators (21.91% versus 13.28%) and NSG CORE indicators (17.41% versus 10.47%) than Regions not under LEA assessment. Much higher percentages characterise the Regions currently under RP (26.60% and 23.38%). Finally, PNE indicators account for 8.73%, with Sicily assigning the highest weight (20%), while five Regions exclude them entirely. Similar to NSG trends, Regions under LEA assessment, and those under RP, assign greater weight to PNE indicators (9.91% and 9.07%) compared to regions not under LEA assessment (4.94%).

####  Measured Performance by Regions 

 These findings verified whether effective incentives guided behaviours towards system-level priorities. Therefore, we assessed regional performance within the national PMSs to examine incentive effects and select the four Regions for further in-depth study in phase two. Regarding the NSG, only eight out of 21 Regions were sufficient across all three healthcare areas during the period investigated. None of these eight were under RP (apart from Piedmont until 2017) or receivership, and none were exempt from LEA assessment (except A.P. of Trento). Among the remaining 13 Regions, four (19.05%) failed in all three healthcare areas, three of these were under RP and receivership and one exempt from LEA assessment. Nine (42.86%) were insufficient in two areas, with five under RP (although Sardinia only in the past) and four under receivership (Abruzzo until 2016 and Campania until 2020). Additionally, two were exempt from LEA assessment. Eleven Regions were insufficient in one healthcare area.

 Overall performance in the NSG and PNE was compared using the two composite indicators described in Table 1. A clear relationship emerged between performance in the two systems. Seven of the eight Regions with sufficient performance across all three healthcare levels demonstrated PNE performance above the sample average, and the seven Regions under RP (two of which are also under receivership) showed PNE performance below the sample average. Figure 2 maps the Regional performance in these two national PMSs using a 2X2 matrix, distinguishing values above and below the sample average.

**Figure 2:**
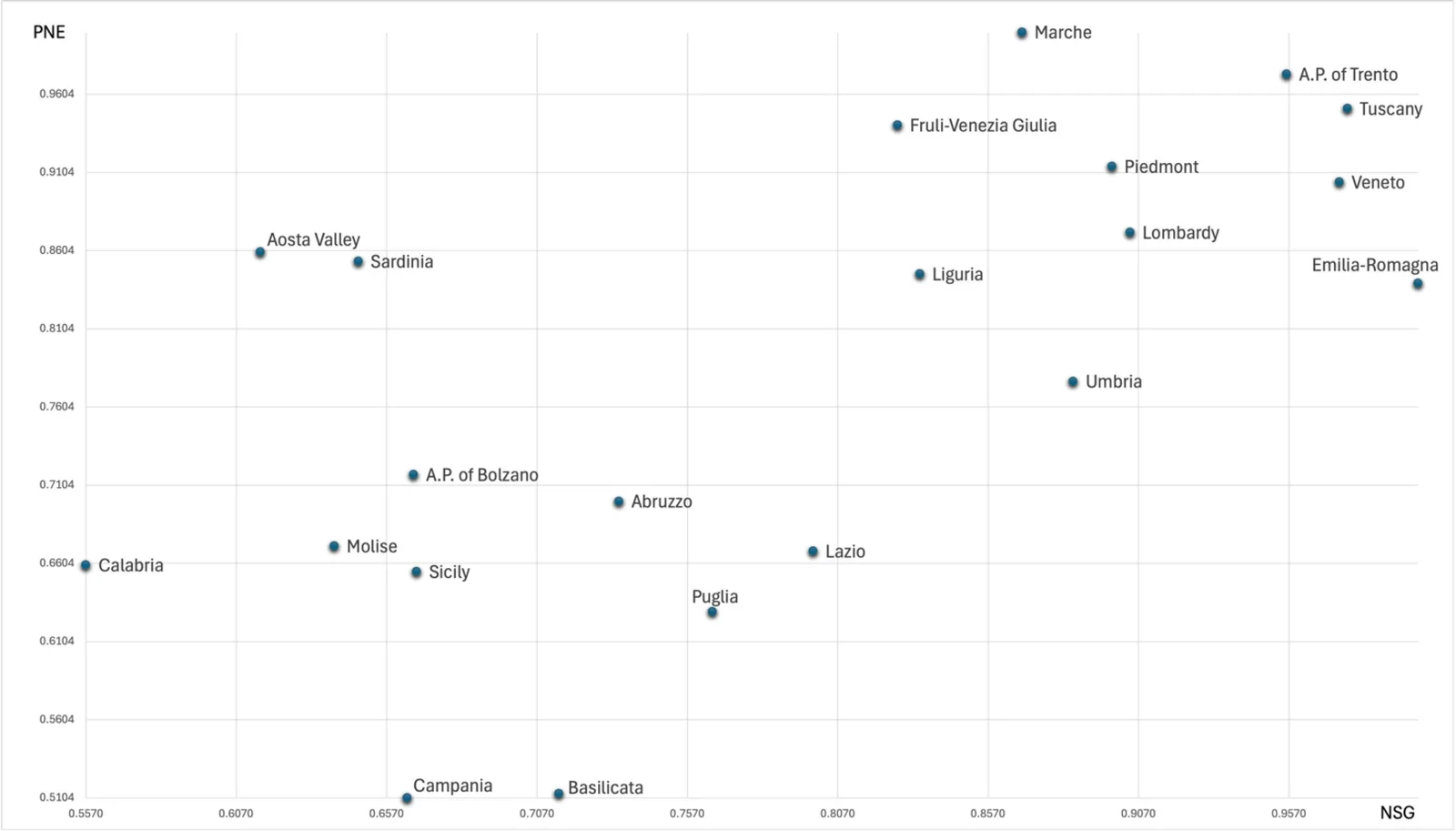
Map of Regions Based on National Performance Measurement Systems (2016-2023). Abbreviations: NSG, Nuovo Sistema di Garanzia; PNE, Programma Nazionale di Valutazione degli Esiti.

###  Qualitative Evidence

####  Aims of Performance Measurement Systems

 Analysing of the aims of the PMSs across the Italian multilevel governance health system reveals the dimensions and actors subject to evaluation, and therefore the overall consistency among the PMSs developed within these systems. The findings indicate that the two national PMSs are complementary: the NSG targets Regions to monitor the provision of LEAs, while the PNE helps hospitals and single units to improve the provision of LEAs and reduce disparities. However, within the INHS an agency problem emerges. While national PMSs focus exclusively on effectiveness and equity, at lower levels PMSs are driven by legal constraints or financial sustainability requirements to prioritise efficiency, appropriateness and safety, highlighting a misalignment between national and regional targets. The healthcare provider organisations are also committed to meeting the population’s health needs while complying with regional regulations and achieving a financial balance. As explained by General Director ‘B’:

 “*The indicators from the PNE and the NSG (...) are health-related indicators, or indicators of care pathways, pathway efficiency, or outcomes. However, there are also some financial indicators selected by the Region. As a healthcare organisation, we obviously need to monitor aspects and areas that we consider to be more strategic or that require closer attention by departments, single units and groups of professionals.”*

####  Incentives

 Although in principle Regions consider both national PMSs equally important, in practice, those under LEA, RP or receivership prioritise NSG. This confirms our findings regarding PMS alignment. At both the regional and organisational levels, financial and autonomy incentives often outweigh reputation, which is generally deemed to be more effective in contexts with mature management cultures and better LEA provision. This sheds new light on the relationship between incentives and culture.^6^ As observed by Executive ‘A,’ from a Region under RP and in receivership:

 “*I can’t say ‘I focus on LEAs and then not monitor the PNE indicators’ (...) However, we prioritise LEAs more strongly and pay much closer attention because we are accountable to the Ministry and are also under receivership. Everything is tied to LEA—rewards, resources—they are essential for us.”*

 The greatest impact of reputational incentives appears to be on health professionals, especially when financial incentives are limited or misaligned with organisational goals (based on which the organisation receives financial rewards) and these rewards are not tied to individual professional contribution.

####  Positive Functional Consequences

 Based on senior executive perception, we identified three key positive functional consequences of PMS in multilevel governance health systems. The first involves simultaneous, multilevel benchmarking of comparative performance. Specifically, the NSG allows regional monitoring and comparison of processes and outcomes, facilitating the identification of best practices and solutions to enhance nationwide, high-quality, equitable care. Meanwhile, the PNE empowers individual organisations to benchmark against standards, pinpointing strengths and weaknesses to adopt successful strategies from high-performing peers. As elaborated by General Director ‘A’:

 “*The assessment of objectives is experienced with trepidation, because healthcare organisations are constantly comparing. To say that we don’t look at other organisations or at the results they achieve would be untrue (…) Also because, at times, when an organisation is recognised for its virtuous performance, there is a direct comparison with that to understand what strategies it has implemented to achieve the objective better than the organisation one is managing.”*

 At the same time, benchmarking fosters improvement through accountability and engagement, which can be summarised as an advocacy function, both in the relationships between Regions and healthcare organisations and between healthcare organisations and professionals. The healthcare organisations commit to achieving the objectives that the Regions will be evaluated against in exchange for appropriate resources, while in turn managers heading organisational units are evaluated on their leadership’s contribution to organisational performance.

 For this framework to function effectively, each Italian region’s PMS must be consistently and firmly aligned with national PMSs. This happens in exemplary cases, as highlighted by General Director ‘D’:

 “*The regional PMS has grown significantly over the years in line with national PMSs, and now represents a valuable source of information for the organisational level as well, particularly for comparing the performance among regional organisations and, through these comparisons, identifying potential areas for improvement.”*

 The PMS also facilitates collaboration between different units and professionals within the organisation as well as with external organisations. As noted by General Director ‘B’:

 “*The indicators serve as unifying elements for those on the front line: they provide a framework for interpretation that various professional roles can converge upon, helping them to understand the reality of what the organisation is doing.”*

 The second positive functional effect is generated at the regional level and is due to the national PMSs offering a comprehensive overview of performance across multiple key dimensions of service delivery, which contrasts with local data systems that may focus on a narrower range of performance metrics. This integrated, holistic approach permits a more holistic overview of how well services are performing and enhances understanding of how different services and teams interact and contribute to overall outcomes.^18-20^

 A benefit highlighted by Regional Executive ‘B,’ who argued:

 “*My impression is that national PMSs are strongly associated and have both a strong impact, albeit in different ways: the release of the PNE draws significant attention from individual professionals, particularly due to reputational aspects, whereas the NSG’s monitoring of the LEAs carries greater weight from a more strategic perspective, in terms of planning interventions and setting high-level directives to be followed.”*

 In Italy, therefore, the two national PMSs not only generate a drive for improvement both for those responsible for ensuring the proper delivery of services, namely the Regions, and for those who actually provide the services, namely the organisations, but they also promote coherence and completeness in evaluation. The Regions that are more culturally advanced are able to focus organisations’ attention on a broad and representative range of aspects linked to high-quality healthcare services, integrating the requirements of the NSG and the PNE into their own PMSs and preserving a balance between coverage and practicality. This makes it possible to steer behaviours and outcomes towards the desired goals, as reflected in the words of Regional Executive ‘B’ and General Director ‘H’:

 “*At the regional level, (…) for years now the objectives we set for organisations have been based on these indicators, so these national systems—the NSG and the PNE—have strong strategic significance.”*

 “*We consider them to the same extent: both tools are certainly very useful for all of us and must be integrated. I see them precisely as complementary. As our Region has done, we too have adopted both, as far as possible — both the NSG and the PNE.”*

 The third positive consequence is the quantification of performance metrics. This was reported to facilitate better information flow between principals and agents at all levels of the system. We heard that reducing complex activities and health outcomes to numerical data minimises subjectivity and provides objective information for comparison. Senior executives argue that these metrics offer concrete evidence of success and failure across Italian regions, fostering a culture of accountability. This is explained by Regional Executive ‘B’ in relation to monitoring the performance of individual physicians:

 “*Especially physicians, before having a PMS, had a distorted perception of reality and saw themselves as the best in their field. The system helps to regulate (though reducing it will be difficult) their self-referential attitude because measures are validated and shared.”*

 At the organisational level, quantified performance metrics were thought to play a crucial role in enhancing strategic decisions, particularly regarding resource allocation. By providing measurable data, these metrics allow organisations to assess the effectiveness of current strategies, identify areas for improvement, and make informed choices about where to invest resources for optimal outcomes. It was reported that the performance metrics provided a framework for evaluating different options and enabled organisational leaders to make more informed decisions based on objective evidence rather than respond to special pleading by pressure groups or professional interest groups. General Director ‘C’ elaborated on this point:

 “*In the past, the typical head physician would walk into the director’s office and say: ‘Listen, Director, if you don’t give me six or seven doctors, or five or six nurses, I’ll be forced to shut down some activities.’ That was the classic statement, which I challenged as much as I could, because I had serious doubts. That’s why objectification is essential: to base decisions on concrete, reliable data.”*

###  Dysfunctional Consequences

 Across the three multi-level agency relationships examined, we found evidence supporting the existence of all 20 dysfunctional consequences (except for “creating a diversion”), previously identified by Mannion and Braithwaite, in the INHS.^2^ Additionally, we also uncovered three new types of dysfunctional consequences: Measurement Overload, Misconsideration, and Exploitation. Below, we provide a more in-depth examination of these three novel consequences. Table 3 explains the meaning assumed by all dysfunctional consequences within the INHS, serving as an example of a multilevel governance health system, and highlights which agency relationships are affected.

**Table 3:** Dysfunctional Consequences in the Italian National Healthcare Service

**No.**	**Type**	**Meaning in the INHS**	Agency Relationships*		
			**1**	**2**	**3**
1	Measurement fixation	Occurs when indicators from national PMSs are used as a control mechanism to set objectives for the senior management of healthcare organisations, and when the media effect of the results prevails over the professionals.		•	•
2	Tunnel vision	The pressure exerted by “controllers” can lead those being monitored to focus solely on the aspects subject to evaluation. It is often associated with measurement fixation.		•	•
3	Myopia	Concerns the inability of the indicators to measure and incentivise medium- to long-term performance and, within organisations, limited feedback to professionals on past results and future strategic directions.	•	•	•
4	Ossification	Concerns the rigidity of behaviour that characterises the actions of healthcare organisations due, for example, to the fact that performance goals tend to remain the same year after year.			•
5	Anachronism	At the national level, information arrives with significant delay and this time lag also appears in the delayed performance evaluation and incentive allocation by Regions, which leads to planning issues and staff demotivation at the organisational level.		•	
6	Quantification privileging	Occurs when reducing services to numbers is not balanced, at all levels, with comparison and the soft information gathered through dialogue with those being evaluated. The multiplicity of governance levels increases workload and the need to address urgent issues, heightening the issue of limited time availability.	•	•	•
7	Overcompensation	Is virtually non-existent due to the lack of financial incentives.	•	•	•
8	Undercompensation	Is virtually non-existent due to the lack of financial incentives. At the organisational level, the lack of financial incentives can create problems related to leadership turnover.	•	•	•
9	Insensitivity	Is associated with the inability to identify and properly reward those responsible for performance and mainly due to a crucial gap at all levels, ie, the poor quality of data.	•	•	•
10	Increased inequality	Differences between Regions and healthcare organisations are limited due to the poor incentives connected to national and regional PMSs, but inequality may increase due to insufficient support actions for those showing low performance.	•	•	•
11	Complacency	Is essentially neutralised by the strong stimulus for improvement generated by benchmarking, advocacy, and collaboration.			•
12	Silo-creation	At the organisational level, can be considered as a side effect of exploitation when evaluated often request a transfer to another high-performing unit in exchange for adopting the behaviours required.			•
13	Misrepresentation	Is caused by the qualitative deficiency of data, which may allow deliberate manipulation of data by staff.	•	•	•
14	Gaming	Occurs only at the organisational level when top management or middle managers, acting as evaluators, deliberately alter assessments in order to avoid organisational conflict.			•
15	Misinterpretation	Is due to a lack of contextualization and dialogue, and amplified by poor data quality. It presents a higher risk of emerging in multilevel systems since the distance between those who steer the system strategically and those who deliver the services increases.		•	•
16	Bullying	Exacerbated by the multiple levels of governance, emerges in organisational contexts deprived of management culture where PMSs are perceived as a form of finger-pointing and may degenerate into punitive and retaliatory tools by middle managers intending to settle personal matters.			•
17	Erosion of trust	Is mainly due to the lack of inclusion of the perspective of citizens and patients in national, regional and organisational PMSs. It is enhanced by the difficulty for users in correctly interpreting the reported data.	•	•	•
18	Reduced staff morale	Can be further reduced in multilevel governance health systems by the concomitant effects of insensitivity, insufficient support and misinterpretation, and is naturally more keenly felt at the organisational level.	•	•	•
19	Political grandstanding	At the national level, it is associated with the manipulative use of information derived from PMSs by politicians and the media, while at the regional level by local politicians and associations.	•	•	•
20	Creating a diversion	It does not arise as a discernible effect from political grandstanding.			
21	Measurement overload	At the regional and local levels, excessive bureaucracy and a proliferation of indicators can lead to inefficient and complex processes with a proliferation of performance indicators to be collected and reported.		•	•
22	Misconsideration	Occurs due to the complexity of the INHS, and the inability to take fully into account all contextual and situational differences, both regional and organisational and the wide range of services that contribute to the quality of healthcare provision.	•	•	•
23	Exploitation	Occurs when performance goals, the behaviours that stem from them, and the associated incentives are misaligned across the various levels that make up multilevel governance health systems, which can inadvertently foster a culture where individuals leverage their positions for personal gain rather than contributing to overall organisational success.			•

Abbreviations: INHS, Italian National Healthcare Service; PMSs, performance measurement systems. * Legenda: 1 = State-regions; 2 = Regions-healthcare organisations; 3 = Healthcare organisations-professionals.

*Measurement Overload*: while the national level can benefit from the use of multiple PMSs to assess diverse aspects of performance, regional and local levels often experience excessive bureaucracy and a proliferation of indicators when implementing multiple systems. This can lead to inefficient and complex processes at the local level, with too many performance indicators to be collected and reported. As noted by General Director ‘B’:

 “*The organisation is measured against 600 performance indicators. There are the NSG indicators, the PNE indicators, the Treemap, then those from the regional model, plus two or three additions we might decide to introduce ourselves: it’s an endless list (…). You can be measured on everything, but you obviously can’t monitor everything, every day.”*

 An overabundance of performance measures can create conflicting objectives, where efforts to improve in one area may negatively impact another, or when organisations struggle to prioritise effectively. As General Director ‘G’ observed:

 “*The Region, with one indicator, tells us to ‘increase production,’ and with another tells us to ‘cut waiting lists,’ and then gives us yet another indicator that says ‘reduce drug prescriptions.’ Things that simply can’t be reconciled with each other.”*

*Misconsideration* occurs due to the complexity and diffuseness of the Italian multilevel governance health system, and the inability to take fully into account all contextual and situational differences, both regional and organisational and the wide range of services that contribute to the quality of healthcare provision. We heard that performance measures to be effective need to account for the interconnectedness of all elements of the health system, as performance is not solely determined by individual components but is shaped by system-wide interactions. As General Director ‘E’ explained:

 “*It’s obvious that all the work we’re doing isn’t being captured, and that, from a certain point of view, is deeply demotivating. It’s true that we should all be assessed by the same standards, but it’s also true that there are discrepancies, different starting points.”*

 At the organisational level, national PMSs often overlook specific clinical areas, causing a rift between policy and practice. This leads physicians to feel that their professionalism is undervalued and suggests that these systems are driven by financial motives rather than their stated goal of improving healthcare. In Italy’s multilevel governance health system, financial constraints on Regions and healthcare organisations further exacerbate this, often resulting in internal units within organisations being assigned objectives entirely disconnected from clinical reality. This tension is clearly captured and reported by a head physician during a focus group discussion:

 “*Last week, the top management of my organisation gave me ten objectives that my team and I are expected to achieve in the coming year. Do you know how many of them relate to the services we provide to patients? Do you really want to know? None! They are all financial and managerial targets. It’s a disgrace! These people neither know nor want to know what we do every single day!”*

 This lack of recognition deeply demotivates professionals and fuels opposition to the PMS. When work and expertise are not acknowledged, either at the national level or organisational level, it can lead to the dysfunctional consequences described below.

*Exploitation* emerged at the organisational level due to misalignments in the PMSs between organisational goals and unit level objectives. These inconsistencies, alongside divergent motivations between managers and professionals, can foster a culture where individuals seek to leverage their positions for personal gain rather than contributing to overall organisational success. This creates a power dynamic that can undermine collective effort.

 Indeed, misalignment between performance goals, incentives and staff behaviours in multilevel health governance can inadvertently erode professionalism. This occurs when senior management uses the PMS to influence practitioners, without accounting for their underlying ethical profiles, as illustrated by a unit head during one of our focus groups:

 “*I really dislike the instrumental way my organisation uses the PMS: my efforts serve to make them look good in regional and national assessments, yet I am not adequately rewarded for the contribution I make every day to improving the quality of care.”*

 When professionals feel their efforts are undervalued or misunderstood, they may adopt coercive or retaliatory stances. This was thought of as manifest by middle managers exploiting the system to their advantage by demanding concessions, such as relaxed targets or increased autonomy from senior managers.

 Conversely, virtuous instances exist where health organisations adapt regional objectives to better align with their operational realities. In these cases, as noted by General Director ‘H,’ the organisation may forgo financial incentives to prioritise professional satisfaction:

 “*I redefine the (regional) target at the organisational level. Because I need to make it, in some way, consistent with our reality. Okay? So, I end up in a situation where, if I adjust it, they (professionals) achieve it, while I won’t. Never mind.” *

 Similarly, health professionals may use their indispensable expertise to negotiate for additional resources in exchange for meeting an organisation’s quantitative targets. This illustrates how poorly designed PMSs can be manipulated and exploited, fostering cultures where personal self-interest is prioritised over collective goals – a sentiment echoed by two focus group participants:

 “*Over the years, many times the head of my unit has explicitly told the General Director that, if he wanted to meet the LEA standards, ‘in the interest of the organisation,’ he would have to give him more resources!”*

 “*Sometimes my colleagues resort to outright blackmail against the head of the unit evaluating them: ‘If I receive a low evaluation, I’ll change department!’ The real problem is that management allows this kind of behaviour.”*

## Discussion and Implications

 In this section, we first analyse the findings in the context of the existing literature. We then discuss policy implications - the ultimate aim of this research - by identifying key effectiveness factors and proposing 10 recommendations to strengthen performance measurement in the INHS.

 Our study responds to the call for deeper investigation into the dysfunctional consequences produced by PMSs across different national regimes and strategies to mitigate them.^48^ Furthermore, we address the gap in research regarding positive unintended consequences, which are often overlooked.^6^

 While our results focus on providers and organisations, they do not show a significant association with the five positive effects identified by Li and Evans.^6^ The sole similarity found relates to “motivated learning and development.” However, in the multilevel governance regime of the INHS, this stems from the capacity to compare performance across regional and local levels. Notably, no improvements in enhanced planning capacity were observed.

 The second positive effect of PMSs in the INHS is “comprehensiveness.” This attribute helps counter issues identified by Powell et al,^47^ which often stem from implementing local strategies to meet national-level demands. In this context, the Regions, positioned between the national and local levels, can benefit from Italy’s dual evaluation system, which enables monitoring across a wide range of dimensions and activities. However, to do so effectively, they must filter and contextualise to steer organisational action toward their specific system goals. This balance between the representativeness and coverage of the regional PMS and its practical role in guiding organisational behaviour is an especially complex task,^26^ and is most evident in Regions with long-standing PMS tradition and where there is a close alignment, in terms of objectives and indicators, with national standards. Finally, this study reveals several new dysfunctional consequences emerging within the INHS that warrant further discussion.

 In multilevel health governance, Measurement overload describes a heightened risk where a proliferation of indicators creates a system too complex for practitioners to interpret and prone to misuse.^26^ Without efforts to rationalise objectives, the presence of an intermediate regional level can obscure goals, drive conflicting behaviours and increase administrative burden through heavier workloads and inconsistent methods for calculating indicators. This burden, defined by Li and Evans as “excessive time spent on administrative tasks,”^6^ is amplified at the organisational level. In the INHS, this may ultimately lead to a deterioration in care performance due to reduced direct patient time,^47^ particularly where the relationship between Regions and health organisations is marked by political conflict.

 Although misconsideration is linked to various dysfunctional consequences in existing literature, it takes on a particular significance in multilevel governance health systems as well. This occurs when PMSs at neither the national nor the regional level adequately capture the value of professional work, often neglecting specific clinical areas entirely. This lack of consideration causes clinicians to view these systems as purely instrumental and driven by financial incentives rather than patient care, as noted by Conrad and Guven Uslu.^24^ In the absence of reputational incentives that typically motivate healthcare professionals, resistance to these purely managerial metrics intensifies.^46^

 This mechanism aligns with the concept of “increased perceived injustice,” a dysfunctional consequence identified by Li and Evans.^6^ This occurs when professionals develop resentment due to social comparisons between groups subjected to PMS and those that are not. As Siverbo et al suggest,^43^ such reactions are predictable when public-sector PMSs rely on measures that service providers find unrecognisable or fail to capture their actual efforts.

 Furthermore, misconsideration can be seen as an extension of “tunnel vision,” which emerges in multilevel governance health systems when, as Aryankhesal et al observe, organisations, driven by an instrumental approach that prevents them from addressing deficiencies in the representativeness of higher-level PMSs, “neglected to focus on some important aspects of quality and performance as these were not measured and rewarded in the grading system.”^48^

 In extreme cases, misconsideration may lead those being evaluated to what Li and Evans term a “systemic dysfunction,”^6^ where performance priorities and measurement methodologies are misaligned or contradictory across hierarchical levels within the broader healthcare system. In fact, individuals and organisations may seek to maximise personal gain at the expense of collective welfare. In Italy’s multilevel health system, this occurs in both the relationships between Regions and health organisations, and between those health organisations and their individual units. This is particularly evident in cases of misalignment between objectives at different system levels,^47,50^ which induces agents (embodied in the INHS respectively by senior management and professionals) guided by goals not aligned with those of the principal (ie, the Region or the organisation’s senior management) to adopt coercive behaviours towards the evaluator. This occurs when agents’ personal goals conflict with those of the principal, such as regional authorities, because agents are responsible for generating the very data used to evaluate them.^26^

 Under these circumstances, PMSs developed in multilevel governance health systems carry an increased risk of eroding professionalism by incentivising physicians to abandon their core principles under the weight of system-imposed costs.^45^

 The key factors that could be used to improve performance measurement in the INHS are outlined in Table 4. Derived from a synthesis of our quantitative and qualitative evidence, these recommendations seek to address multi-level agency issues, specifically by reinforcing beneficial consequences while mitigating adverse effects.

**Table 4:** Effectiveness Factors of Performance Measurement System in the Italian National Healthcare Service

**No.**	**Type**	**Meaning in the INHS**	**References**
1	Incentive alignment	Incentives within the healthcare system need to be aligned across all levels – State, Regions, and individual healthcare organisations – to ensure a common goal of improved service delivery and adherence to national standards. While financial incentives are currently limited at all three levels, the motivation of frontline professionals could be strengthened by adjusting the incentive structure for specific goals and roles (variable geometry). This approach can be more effective than relying on ethical or reputational incentives, particularly in organisations with less mature management cultures.	^5,9,16,17,24,50^
2	Indicator consistency	There is a pressing need for better integration of national indicators into regional PMSs to create a consistent framework for evaluating performance across different Regions and to develop targeted interventions in areas where certain regions might need additional support and assistance. Such an approach can facilitate the sharing of best practices from high-performing Regions and organisations to those requiring improvement	^6,26,47^
3	Professional training	Investing in the training of professionals to improve data quality, with beneficial effects on the reliability and use of PMS information at national and regional levels. For instance, in the INHS, training programmes at the organisational level aimed at equipping staff with the skills to accurately record and classify clinical information related to hospital admissions (ie, the coding of hospital discharge records) should be developed. This would address major data quality concerns and foster a culture that values data-driven insights. It would also compensate for the lack of dedicated education in clinical data management and information governance in traditional healthcare management curricula.	^26,33,51^
4	Information system interconnection	Implementing stronger system-level control over data through the interconnection of information systems at national, regional, and organisational levels of the INHS. The creation of a central platform and interoperable systems between State, Regions, and health organisations would significantly enhance data reliability and consistency within the INHS. This centralisation of data flows would improve data management and utilisation, fostering collaboration and improving data integrity across different tiers of governance.	^31,52^
5	Timeliness	The PMS should be designed to provide relevant stakeholders with timely, accurate, and actionable information, facilitating swift decision-making and prompt action when required. Therefore, national PMSs should provide data relating to activities that are not too distant in time, in order to enable improvement actions. At local level, the most efficient Regions and healthcare organisations in the INHS have developed advanced PMSs that ensure data integrity and reliability through robust data collection and validation procedures. Furthermore, Regions should proceed more quickly with closing the performance cycle, providing incentives to health organisations in a timely manner, so as not to undermine the credibility of the system and the motivation of those delivering the services.	^24,53^
6	Support actions	Initiatives to spread best practices and support remediation pathways following evaluation should be expanded at both the national and regional levels. At the moment, in the INHS, at the national level audits are activated only after PNE evaluation whereas many regions still do not provide support measures for health organisations that demonstrate low performance. In resource-scarce settings, organisations may lack the necessary in-house expertise or capacity to address complex problems. Collaboration with external bodies and organisations can facilitate the sharing of successful strategies from high-performing Regions and organisations to those requiring improvement in performance.	^4,34,54^
7	Flexibility	Sub-national PMSs, those developed by Regions and health organisations, should be made more flexible and tailored to specific contexts and diverse patient populations. Multilevel governance configurations can address the shortcomings of national and regional PMSs by fostering broader representation of clinical areas. In this regard, regional and healthcare organisations’ PMSs should adapt existing indicators and introduce new ones to capture dimensions not covered by national or regional PMSs, ensuring more comprehensive performance evaluation. The engagement of healthcare organisations and middle management is crucial for the design of more sensitive and comprehensive PMSs.	^6,21,23,24,26,36,42,45,46,48,55^
8	Dialogue	More frequent and open dialogue between different levels of the system, for instance, between the Ministry of Health and the Regions and the Regional health authorities and the health organisations, would promote a better understanding of roles, responsibilities and challenges across the system. This would enhance PMSs’ ability to accurately represent the nuances and complexities of everyday operations and clarify the interconnectedness of different system components, fostering a more holistic understanding. Increased opportunities for discussion and information exchange should also allow for the sharing of soft information between evaluators and those being evaluated, thereby helping to clarify the reasons behind observed results and highlighting contributing factors and qualitative insights that may not be immediately apparent in quantitative metrics.	^26,32,37,38,56,57^
9	Patient engagement	Incorporating citizens’ and patients’ perspectives is crucial for comprehensive performance assessment, as such an approach will offer unique insights into service quality and areas needing improvement and attention that are often missed or overlooked by those working at different levels of the system. This means actively seeking input from patients and citizens on their needs, preferences and expectations regarding their care and incorporating this feedback into the design and implementation of PMSs. At the moment, in the INHS, the opinion of service users and the community is not adequately considered in either national or regional PMSs.	^6,21,22,24,35,40,41,47^
10	Literacy	To combat potential trust erosion in local health services due to poor local performance or misinterpretations of information, it is crucial to take a more proactive approach to developing communication strategies and training for both the public and healthcare professionals. This would involve disseminating performance information in plain language format that avoids jargon and initiatives to enhance health literacy among the general public. In the INHS, indeed, although the information produced by national PMSs is disseminated (and in the case of the PNE, even in great detail through the online channel), and in some cases the results of regional PMSs as well, there is a substantial difficulty on the part of citizens in correctly interpreting the data. For this reason, training is also required to ensure that general practitioners are well-prepared with the skills to interpret and communicate performance data effectively to patients and to provide appropriate guidance when required.	^15,26,33^

Abbreviations: INHS, Italian National Healthcare Service; PMSs, performance measurement systems; PNE, Programma Nazionale di Valutazione degli Esiti.

## Conclusion

 This study assessed the functional and dysfunctional impact of PMSs in the Italian multilevel governance health systems. We identified three key functional consequences. First, PMSs drive improvement by facilitating benchmarking, advocacy and collaboration, and facilitate better-informed decision-making. Second, quantifying activities and services enhances objectivity and transparency, allowing the identification of successful strategies and best practices. Third, national PMSs offer a comprehensive view of performance across multiple dimensions and provide a more holistic understanding of how different aspects of the system function and interact. We found a wide range of dysfunctional consequences induced by the PMSs, and our analysis led to the identification of three new dysfunctional consequences in the Italian context: Measurement Overload, Misconsideration, and Exploitation. Based on these results, set alongside insights from the international literature, we outline 10 effectiveness factors that could be used to improve and refine PMSs in the Italian system.

 This study contributes valuable insights for optimising relationships between different levels of government, thereby enhancing management and service delivery within multi-level healthcare systems. Despite offering novel evidence on the functional and dysfunctional consequences of PMSs in the Italian context, our findings are context-dependent, warranting further research to validate these factors across multiple health systems with different structures and regulations.

## Disclosure of artificial intelligence (AI) use

 Not applicable.

## Ethical issues

 The research involved human participants. All authors certify that participants decided to take part in the analysis voluntarily and provided informed consent to participate. They consented to participate in the analysis and to be recorded in line with GDPR 2016/679 EU and Decree no. 196/2003. Participants (Regions, healthcare organisations and professionals) were granted total anonymity and were adequately informed of the aims, methods, institutional affiliations of the researchers, and any other relevant aspects of the study. In line with the Helsinki Declaration and the Italian legislation (acknowledgement of EU Regulation no. 536/2014 on January 31, 2022 and Ministerial Decree of November 30, 2021), ethical approval by a committee was not required since the study was non-medical and non-interventional.

## Conflicts of interest

 Authors declare that they have no conflicts of interest.

## Disclaimer

 The authors certify that the manuscript comprises original and unpublished materials that are not under consideration for publication elsewhere. They also declare that the views expressed in the submitted article are their own and not an official position of the institutions (or funder, since the research did not receive funds).
